# Person identification from aerial footage by a remote-controlled drone

**DOI:** 10.1038/s41598-017-14026-3

**Published:** 2017-10-19

**Authors:** Markus Bindemann, Matthew C. Fysh, Sophie S. K. Sage, Kristina Douglas, Hannah M. Tummon

**Affiliations:** School of Psychology, University of Kent, Canterbury, UK

## Abstract

Remote-controlled aerial drones (or unmanned aerial vehicles; UAVs) are employed for surveillance by the military and police, which suggests that drone-captured footage might provide sufficient information for person identification. This study demonstrates that person identification from drone-captured images is poor when targets are unfamiliar (Experiment 1), when targets are familiar and the number of possible identities is restricted by context (Experiment 2), and when moving footage is employed (Experiment 3). Person information such as sex, race and age is also difficult to access from drone-captured footage (Experiment 4). These findings suggest that such footage provides a particularly poor medium for person identification. This is likely to reflect the sub-optimal quality of such footage, which is subject to factors such as the height and velocity at which drones fly, viewing distance, unfavourable vantage points, and ambient conditions.

## Introduction

Unmanned aerial vehicles (UAVs), commonly referred to as drones, are increasingly utilised by police and the military. In the UK, for example, a key application of drones by police organisations is to assist in searches for missing persons^1^, as well as crowd control^2^. In addition, drones are routinely used by the military in operations such as reconnaissance, target acquisition, and to carry out lethal strikes^3,4^. These deployment strategies imply that drone-captured footage provides sufficient information for person identification. However, due to the variable height and velocity at which drones fly, such footage is likely to be subject to momentum, unfavourable vantage points, and unpredictable ambient conditions. For example, military drones operate from ground level up to maximum altitudes of 200 ft for micro drones^4^, which are small tactical drones of up to 2 kg in weight, and up to 45,000 ft for large drones^3,4^ that comprise unmanned long-endurance aircraft of over 600 kg. Moreover, the ground speed at which these drones operate varies considerably, from 0–250 kts^3,5^. In addition, drones employed in police operations record surveillance footage whilst operating at altitudes ranging from ground level to up to 400 ft, and at speeds of up to 38 kts^6^. This range in operational parameters raises the possibility that drone-captured footage can be of sub-optimal quality for person identification.

The current study reports four experiments that investigate this issue, by examining the accuracy of person identification from drone-captured footage of a football (soccer) match at a UK university. This set up presents a natural scenario that should provide relatively favourable conditions for image capture and subsequent person recognition. We recorded such footage with a commercially available remote-controlled drone, with a minimum take-off weight (MTOW) of 300 g. As classified by NATO regulation, this type of drone falls into Class I(b)^7^ and is therefore comparable to micro drones in use by the UK military^3^ and police force^6^. We present four experiments that utilised the footage recorded with this drone to examine the accuracy of person identification from such surveillance material.

To our knowledge, these experiments represent the first systematic investigation of person identification by human observers from aerial footage recorded by a remote-controlled drone. By contrast, a compelling body of research already exists on person identification in other applied settings, such as passport control^8,9^, closed-circuit television (CCTV)^10,11^, and eyewitness scenarios^12,13^. This research demonstrates that *familiar* people, who are known to an observer, can be identified with good accuracy^14,15^. This is found under challenging conditions, for example, when people are viewed in poor-quality surveillance footage^11^, or heavily degraded video^16^, or when they are only seen briefly^17^, partially^18,19^, or in unfavourable non-frontal views^20^.

This reliable recognition of familiar people is held to be based on sophisticated cognitive representations that build up through substantial exposure to a person’s face across a range of ambient conditions^21,22^. Such experience enables the extraction of the stable visual characteristics of an individual’s identity, and for the dissociation of this information from ambient factors that interact with a person’s appearance, such as variation in lighting or viewing direction^23^. The exact nature of these representations remains under investigation, but might reflect a cognitive “average” of the encounters with a face^22,24^, with dimensions that capture the different ways in which a person’s appearance can vary around such an average^21,25^. Such approaches view familiarity as a continuum, from unknown to well-known faces. Consequently, whether a specific point exists on this continuum at which faces can be defined as “familiar” is an open question. What is clear, however, is that when the cognitive representations of familiar faces are firmly established, these allow for recognition to generalise across a broad range of conditions, and to succeed even with very limited visual information^11,18,19^.

By contrast, the identification of unknown or unfamiliar people, of whom an observer has no prior experience, is error-prone, even under seemingly good conditions. For example, when observers try to identify a target from a ten-face array, accuracy is only at 70%^10,26^. This is found with high-quality images that depict people in a frontal view, with a neutral expression, and under good lighting. Performance remains poor when this task is reduced to a 1-to-1 comparison^27,28^, or when observers match a live person to their photo^29,30^, or moving video images^31^. This difficulty of unfamiliar person identification reflects the fact that, without extensive prior exposure, observers can only have limited information about how a person can vary naturally in their appearance. Consequently, attempts to identify an unfamiliar person have to rely on unsophisticated image-comparison techniques. This issue is illustrated by the fact that unfamiliar face identification is trivial across identical images^21,22^, but becomes more error-prone as variability in a person’s appearance increases across to-be-compared images^32,33^. Similarly, accuracy declines when lighting or viewing angle are variable across images^34^, or image resolution is poor^35^. Considering that drone-captured footage is restricted by such factors, the question arises also of the extent to which unfamiliar people can be identified from such material. In this study, we investigate these questions across several tasks to examine the identification of unfamiliar (Experiment 1) and familiar people (Experiment 2 and 3), as well as the perception of a person’s sex, race, and age from drone-captured footage (Experiment 4).

## Experiment 1

In this experiment, observers were presented with arrays comprising a high-quality face photograph and drone-captured images of a person, and had to decide whether these materials depicted the same person or two different people. Such identity-matching tasks have been used extensively in forensic face identification^36,37^, and minimize the contribution of other factors, such as memory demands, that can reduce performance^38^. In light of the expected difficulty of this task, three drone-captured images were provided for comparison with each face photograph to increase the possibility that correct identifications are made^32,39,40^. Our drone was also equipped with two different forward-facing cameras, the footage of which was compared on a between-subject basis.

## Method

### Participants

Forty students (34 female) from the University of Kent, with a mean age of 22.1 years (SD = 8.0), participated for course credit. All experiments reported in this paper were approved by the Ethics Committee in the School of Psychology at the University of Kent and conducted in accordance with the ethical guidelines of the British Psychological Society. In all experiments, informed consent was obtained from all participants before taking part.

### Stimuli

A remote-controlled Parrot AR Drone 2.0 Power Edition, with a minimum take-off weight (MTOW) of 300 g, was employed for stimulus capture. This type of drone falls into Class I(b) with a MTOW of 200 g-2 kg as classified by NATO regulation^7^, and is comparable to drones that are in use by the UK military^3^ and police force^6^. The drone was equipped with two forward-facing cameras, comprising the drone’s integrated HD camera with a maximum video resolution of 1280 × 720 pixels at 30 fps, and a retro-fitted GoPro Hero4 Silver with a maximum video resolution of 2704 × 1520 pixels at 30 fps. To provide stimulus footage for the experiments, this drone recorded the protagonists of a football game from pitch-side. Maximum flight-height was restricted to 15 metres of elevation using the drone’s navigation software (AR.FreeFlight2.4 v2.4.22). From each camera, a total of 42 images were extracted manually with graphics software, comprising three images for each of 14 different players. This footage was synchronized across cameras, so that it captured the players at the same point in time, but varied depending on each camera’s characteristics. The sets of three same-person images were arranged side-by-side, and displayed each at a size of 150 × 150 pixels for the drone camera at 72 ppi. The GoPro images were presented at a slightly smaller size of 120 × 120 at 72 ppi due to the higher resolution of this recording equipment. In addition, a high-quality full-face photograph was also taken for each player at a distance of approximately 1 m immediately prior to the drone recording. These images were then cropped to remove extraneous background and resized to 250 (W) × 340 (H) pixels at a resolution of 72 ppi.

**Figure 1:**
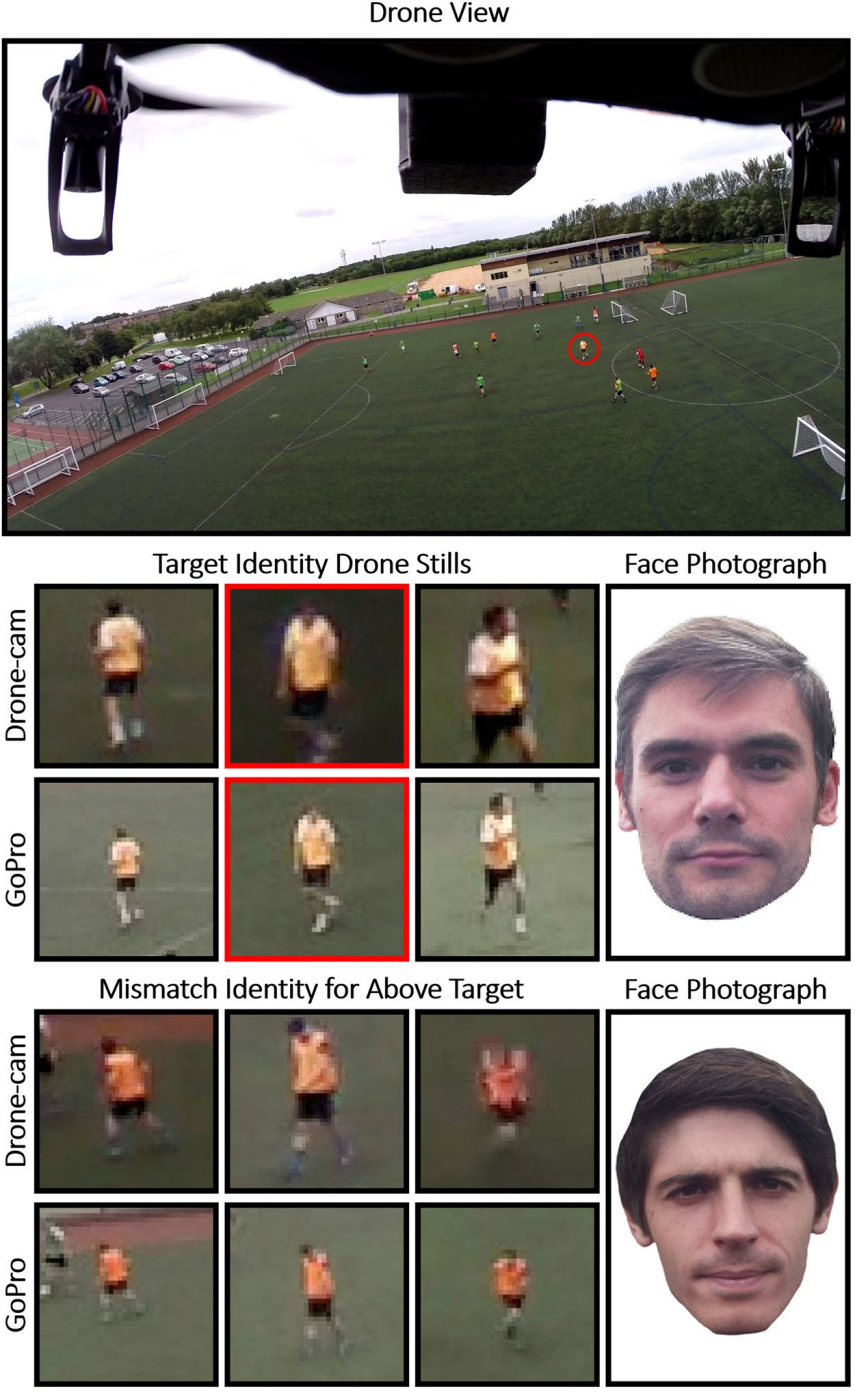
Illustration of an aerial view from the GoPro camera (top) with a highlighted target (red circle). The top array depicts three image stills from the drone-integrated camera and GoPro for this target, and the high-quality face photograph for the familiarity check. The bottom array depicts the corresponding images of the mismatch identity that was selected for this target. Please note that the depicted target, and all other players visible in this figure, have provided informed consent for publication of these images.

To create the stimulus displays, the high-quality full-face images were arranged above three drone-captured video stills. For each of the 14 players, an identity match was created, in which the full-face photograph and drone-captured images depicted the same person, and an identity mismatch, in which two different people were shown. These mismatch pairings were generated by the experimenters (MB and MCF) based on the extent to which different identities were similar in terms of race, hair colour, and age. However, considering the small pool of targets, the number of possible pairings was restricted greatly (for example, the pool of targets comprised only two players of African ethnic origin). Combining the stimulus images in this way resulted in a total of 56 experimental trials, comprising 28 for each drone camera (14 identity matches and 14 mismatches). Example stimuli are illustrated in Fig. 1.

### Procedure

Participants were allocated randomly to one of the two camera conditions. The experiment was run on a computer using *PsychoPy* software^41^. Each trial began with a 1-second fixation cross, which was presented in the centre of the screen. This was followed by a stimulus array, which remained onscreen until a button-press response had been registered. Participants were asked to decide as accurately as possible whether a stimulus display depicted an identity match or mismatch, by pressing one of two designated buttons on a standard computer keyboard. Each participant completed 28 trials, which were presented in a unique random order.

The matching task was followed by a familiarity check to eliminate stimulus identities that might have been known to a participant prior to the experiment. For this purpose, the high-quality full-face photographs were presented individually and participants indicated whether they were familiar with a target, by providing a name or uniquely-identifying semantic information.

### Data Availability

The experimental stimuli and the datasets generated and analysed during the current experiments are available from the corresponding author on reasonable request.

## Results

The familiarity check indicated that participants were familiar on average with 1.6 targets (SD = 1.2) in the drone camera condition and 0.3 targets (SD = 0.6) in the GoPro camera condition prior to the experiment. As each identity featured in one match and two mismatch trials, this led on average to the exclusion of 4.8 (SD = 3.6) and 0.9 (SD = 1.7) trials in these conditions, respectively. For the remaining data, the percentage accuracy for identity match and mismatch trials was calculated.

For the drone’s integrated camera, match and mismatch accuracy was at 48.4% (SD = 12.9) and 73.2% (SD = 14.1), respectively. Similarly, accuracy for GoPro images was at 37.1% (SD = 16.0) for match trials and 66.7% (SD = 14.4) for mismatch trials. A 2 (camera type: drone cam vs. GoPro) × 2 (trial type: match vs. mismatch) mixed-factor ANOVA of these data revealed a main effect of camera type, *F*(1,38) = 12.94, *p* < 0.001, *ƞ*_*p*_^2^ = 0.25, due to overall higher accuracy for the drone camera, and a main effect of trial type, *F*(1,38) = 50.59, *p* < 0.001, *ƞ*_*p*_^2^ = 0.57, due to higher accuracy for mismatch trials. An interaction between factors was not found, *F*(1,38) = 0.39, *p* = 0.53, *ƞ*_*p*_^2^ = 0.01.

As accuracy was low, this was also compared to chance performance (i.e., of 50%) via a series of one-sample t-tests (with *alpha* corrected at *p* < 0.0125 [i.e., 0.05/4] for multiple comparisons). This revealed that mismatch accuracy for the drone camera and the GoPro was above chance, *t*(19) = 7.36, *p* < 0.001 and *t*(19) = 5.19, *p* < 0.001, respectively. By contrast, match accuracy was at chance for the drone camera, *t*(19) = 0.57, *p* = 0.58, and below chance for the GoPro, *t*(19) = 3.61, *p* < 0.01.

## Discussion

Observers’ ability to match drone-captured images to high-quality photographs of unfamiliar faces was at or below chance, with accuracy averaging 43% across camera conditions, which indicates that positive person identifications could not be made reliably. Mismatch decisions were comparatively better but still highly error-prone, averaging at 70%. This low accuracy was obtained despite the provision of three drone-captured images for comparison with each target, which should facilitate person identification^32,39,40^, and under conditions in which the mismatch stimuli were constructed from a limited number of identities.

As a small extension of this work, we also compared person identification for footage from two different camera types, comprising the drone’s integrated HD camera and a retro-fitted GoPro Hero4 Silver. This revealed an advantage for the drone’s integrated camera (61%) over the GoPro (52%). The difference in identification accuracy between these cameras might reflect that the drone’s integrated equipment is better optimized for the viewing conditions that are incurred by aerial recordings. However, even for footage captured with the drone’s integrated camera, identification accuracy was generally low. By comparison, in face-matching studies that combine high-quality face portraits from more conventional footage in 1-to-1 comparisons, and utilise more refined identity mismatches, mean accuracy is typically at 80–90%^27,28^. The current results therefore suggest that the identification of unfamiliar people from drone-captured footage is a particularly difficult task.

## Experiment 2

Whereas unfamiliar face identification is error prone, recognition of familiar faces, that we have encountered many times before, is much more accurate^42,43^ and proceeds even under challenging conditions, such as when poor-quality surveillance footage is employed^11^. Consequently, it is possible that people can be identified reliably from drone-captured footage when they are familiar to the observer. This was explored in Experiment 2 by assessing recognition accuracy of observers that personally knew the people in this footage. Two groups of observers were compared, comprising colleagues of the depicted targets and members of the same football group. Participants in the latter group had not been present during the recording of the drone footage, but had the additional contextual advantage of knowing who comprised the members of the football team to facilitate identification. Non-face objects can be identified in familiar contexts from images with very low resolution^44^. Experiment 2 investigates whether a similar advantage is also present during person identification from drone-captured footage.

## Method

### Participants

The group of colleagues comprised 17 academic staff members (eight male) at the University of Kent, with a mean age of 37.6 years (SD = 11.0), who worked alongside several of the people that were depicted in the drone-captured footage. The group of teammates consisted of ten participants (all male), with a mean age of 44.8 years (SD = 14.6), who were members of the same football group but were absent from play on the day of the drone recording.

### Stimuli and Procedure

The stimuli were the same as in Experiment 1, but the face-matching task was replaced with a recognition test. Thus, the three-image arrays of drone images were now presented without the high-quality face image and participants were asked to name the depicted people directly. If participants indicated familiarity but were unable to name the target, then they were asked to provide unique semantic information to confirm identification. In this manner, all participants were presented with 28 stimulus arrays, comprising a three-photo array for each of the fourteen target identities and each of the two cameras. After completion of this task, all participants were given a familiarity check comprising the high-quality full-face photographs.

## Results

In the familiarity check, participants recognized on average 3.8 (SD = 0.5) of 14 targets in the colleagues group, equating to 27.3% (SD = 3.8) of identities, and 10.3 (SD = 3.2) of 14 targets, or 73.6% (SD = 22.8), in the teammates group. For these familiar identities, performance with the drone-captured images was analysed by grouping responses into correct identifications of a target (hits), incorrect identifications of a target as somebody else (misidentifications), and those cases in which no identifications were made (misses). In addition, performance was calculated for targets that observers indicated as unknown in the familiarity check. For these, the percentage of trials was calculated on which an identification was incorrectly made (false positives) from the drone-captured images.

The mean percentages of responses that fall into each of these categories are illustrated in Table 1 for both cameras and participant groups. These data show that recognition performance was extremely poor. For example, across both cameras in the teammates group, targets could be identified on only 36% of trials (hits). By contrast, 27% of familiar faces were misidentified as someone else, and 19% of unfamiliar faces were also falsely identified as someone familiar. This poor performance was even more marked in the colleagues group, where hits averaged across both cameras were very low, at 8%, whilst almost twice as many misidentifications (16%) were made.

**Table 1:** Person Identification Performance for Experiment 2 and 3, by Participant Type (Colleagues versus Teammates) and Camera Type (Drone versus GoPro Camera). Standard deviations are shown in parentheses.

Experiment 2	Colleagues			
	*Hits*	*Misids*	*Misses*	*False positives*
Drone camera	11.2 (16.7)	13.4 (20.3)	75.4 (29.4)	14.1 (18.0)
GoPro camera	5.6 (10.4)	19.1 (27.3)	75.3 (29.3)	16.4 (21.2)
	**Teammates**			
Drone camera	36.5 (11.0)	25.8 (17.9)	37.7 (12.6)	18.1 (20.9)
GoPro camera	34.6 (12.9)	28.1 (16.3)	37.3 (17.1)	20.0 (26.2)
**Experiment 3**	**Teammates**			
Drone camera	32.7 (16.1)	8.7 (14.0)	32.7 (16.1)	30.7 (36.0)

To analyse these data, separate 2 (group: teammates vs. colleagues) × 2 (camera type: drone cam vs. GoPro) mixed-factor ANOVAs were conducted for each of the four measures. For hits, this analysis revealed a main effect of group, *F*(1,25) = 37.51, *p* < 0.001, *ƞ*_*p*_^2^ = 0.60, due to higher recognition accuracy among teammates than colleagues. In turn, a main effect of group was also found for misses, *F*(1,25) = 17.21, *p* < 0.001, *ƞ*_*p*_^2^ = 0.41, as the teammates were less likely to fail to identify a known person. For hits and misses, a main effect of camera was not found, *F*(1,25) = 1.67, *p* = 0.21, *ƞ*_*p*_^2^ = 0.06 and *F*(1,25) = 0.00, *p* = 0.95, *ƞ*_*p*_^2^ = 0.00, and no interaction between factors, *F*(1,25) = 0.42, *p* = 0.52, *ƞ*_*p*_^2^ = 0.02 and *F*(1,25) = 0.00, *p* = 0.97, *ƞ*_*p*_^2^ = 0.00, respectively. None of the main effects or interactions were significant for misidentifications and false positives, all *Fs*(1,25) ≤ 2.33, all *ps* ≥ 0.14, all *ƞ*_*p*_^*2*^ ≤ 0.09.

The different response categories were also compared directly to determine which identification outcome was most likely. For this analysis, the data for both cameras were combined and a series of paired-sample t-tests were conducted to compare hits, misses, misidentifications and false positives (with *alpha* corrected at *p* < 0.008 [i.e., 0.05/6] for multiple comparisons). For teammates, this analysis failed to find differences between any of the measures, all *ts*(9) ≤ 2.05, all *ps* ≥ 0.07. Thus, teammates were as likely to make a correct identification as an incorrect identification, or to fail to recognize a target altogether. In the colleagues group, observers recorded more misses than hits, misidentifications and false positives, all *ts*(16) ≥ 5.26, all *ps* < 0.001, whereas these three measures did not differ from each other, all *ts*(16) ≤ 1.95, all *ps* ≥ 0.07.

## Discussion

Teammate observers already knew more of the targets than colleagues prior to the experiment. They were also more likely to identify these familiar targets from the drone-captured footage, and less likely to fail to recognize a known person, indicating a context advantage that facilitated identification from low-quality images^44^. Generally, however, recognition accuracy was poor for both groups. For example, teammates only identified 36% of targets that could be recognized from the high-quality images, and recognition accuracy for colleagues was at just 8%. Thus, still images from drone-captured footage only allow for very limited recognition of familiar people. This problem is compounded by incorrect identifications, both for targets that were personally familiar and unfamiliar, which occurred as frequently as correct identifications.

Once again, we also compared person identification from footage captured by the drone’s integrated camera and a retro-fitted GoPro. As in Experiment 1, an advantage in correct identifications, and a corresponding reduction in incorrect identifications, was obtained for the integrated camera. While this is consistent with the notion that the characteristics of this equipment might be more optimized for aerial footage than the GoPro, these differences were small (~4%) and not statistically reliable in Experiment 2. This might suggest that differences in recording equipment exert less of an effect on the identification of familiar than unfamiliar faces.

## Experiment 3

Whilst the identification of familiar people is difficult from drone-captured still images, identification can be enhanced when moving images are provided^45^, particularly under difficult viewing conditions^16,46^. Experiment 3 therefore investigated the recognition accuracy of familiar people from moving drone-captured footage, by replacing the image arrays of Experiment 2 with 10-second video recordings from which these still images were originally taken. Due to the comparable performance across camera types in Experiment 2, only footage from the drone’s integrated camera was employed in Experiment 3.

## Method

### Participants

The participants consisted of 16 males who were members of the football group that was recorded by the drone. Seven of these participants are depicted in the drone footage, whereas the other nine were absent from play on the day of the drone recording. One participant failed to record their age. The remaining participants had a mean age of 42.7 years (SD = 10.7). Data collection was conducted online and participants were invited to take part via a football members email list.

### Stimuli and Procedure

The stimuli and procedure were identical to Experiment 2, except that the stimulus arrays comprising three drone-captured images were replaced with the video footage from which these still images had been taken. This footage was displayed in an online browser using Qualtrics survey software. In total, 14 video clips were shown, comprising a 10-second recording from the integrated drone camera for each of the 14 target identities. As the recording captured a game of football, several targets were visible in each video clip. The first second of each video therefore displayed a still image in which the to-be-identified target identity was highlighted with a red circle, followed by nine seconds of moving footage that followed on naturally from the still. Following each video, participants were asked to name the target or to provide unique semantic information for identification. The videos were shown in a random order. After completion of the video task, all participants were given the familiarity check comprising the high-quality full-face photographs.

## Results

The familiarity check indicated that participants recognized on average 10.5 (SD = 2.5) of the 14 targets, equating to 75.0% (SD = 18.1) of identities. For these familiar identities, performance with the drone-captured footage was broken down into hits, misidentifications and misses (see Table 1). In addition, performance for unfamiliar targets was also converted into false positives. Note that two of the 16 observers recognized all of the target identities in the familiarity check. Analysis of false positives is therefore based on N = 14.

To determine which identification outcome was most likely, the different response categories were compared directly via a series of paired-sample t-tests (with *alpha* corrected at *p* < 0.008 [i.e., 0.05/6] for multiple comparisons). This analysis revealed that more hits than misidentifications of familiar targets were made, *t*(15) = 4.71, *p* < 0.001. By contrast, the percentage of hits was comparable to false positive identifications of unfamiliar targets, *t*(13) = 0.21, *p* = 0.84. Target misses exceeded misidentifications, *t*(15) = 5.99, *p* < 0.001. Misses also exceeded hits and false positives, but these differences were not significant, *t*(15) = 2.87, *p* = 0.01 and *t*(13) = 1.89, *p* = 0.08, respectively. Misidentifications did not differ reliably from false positives, *t*(13) = 2.27, *p* = 0.04.

To examine the potential benefit of moving footage for identification directly, these data were compared with the teammates’ performance with still images from the drone camera in Experiment 2 via a series of independent-samples t-tests (with *alpha* corrected at *p* < 0.013 [i.e., 0.05/4] for multiple comparisons). This revealed that hits were comparable for still images and moving footage, *t*(24) = 0.65, *p = *0.52, as were false positives, *t*(22) = 0.99, *p* = 0.33. By contrast, still images gave rise to more misidentifications, *t*(24) = 2.73, *p* < 0.013, and fewer misses, *t*(24) = 2.71, *p* < 0.013.

Due to the restricted subject pool that teammates provide, seven of the participants of Experiment 3 also appeared in the stimulus footage as football players. As a final step of the analysis, this allowed us to probe self-recognition from the drone footage. All recognized themselves in the familiarity check, but only three of these seven participants (41.9%) recognized themselves in the drone video. Of the remaining four participants, one misidentified themselves as another person (14.2%) and three could not make an identification (41.9%).

## Discussion

The percentage of correct identifications from moving drone-captured footage in this experiment was comparable to the static drone footage of Experiment 2. Still images gave rise to more misidentifications of familiar people and fewer cases in which no identification was made. This suggests that moving drone footage might lead participants to exert more caution in committing to an identification. At the same time, false identifications, of targets that were not known to participants prior to the experiment, were comparable across both types of footage.

Overall, these data confirm that person identification from drone footage is highly error-prone. This appears to be the case under conditions that typically facilitate identification, namely when recognition of familiar people is examined^11,42,43^, context limits the number of possible answers^47^, and moving footage is supplied^16,46^. In addition, the stimuli also provided body information, which can aid identification further^48,49^. The difficulty of this task is illustrated further by the observation that only three of seven participants who were featured as stimuli could identify themselves from the drone footage.

## Experiment 4

Considering that identification is highly error-prone, the question arises of whether other person information can be gleaned from drone-captured footage. In Experiment 4, observers unfamiliar with the targets depicted in the drone-captured footage were asked to judge the sex, race and age of these persons. This information is typically extracted accurately from high-quality images^50–54^. It is unknown to which extent this is possible from drone-captured footage.

## Method

### Participants

A total of 60 participants (33 female) volunteered to participate in this experiment. Two participants did not record their age. The remaining participants had a mean age of 26.4 years (SD = 15.0). All reported normal (or corrected-to-normal) vision. Data collection was conducted online and participants were invited to take part via an email list for research volunteers.

### Stimuli and Procedure

For each target, a drone-captured still image was selected from the stimulus arrays of Experiment 2, which depicted the person in frontal or near-frontal face view, as well as the high-quality photographs. The drone-captured images were presented at a size of 150 × 150 pixels, whilst digital photographs were presented at a size of 400 × 300 pixels at a resolution of 72 ppi. In the experiment, these stimuli were displayed in a web browser using Qualtrics software on a between-subject basis. Thus, half of the participants viewed the drone images, whilst the other half the viewed high-quality face photographs. For each target, observers were required to make sex, race, and age judgements, which were presented in multiple-choice format. For sex, the response options consisted of “male” and “female”. For race, these choices comprised “White”, “Black”, “Asian”, “Mediterranean”, “Indian”, and “Hispanic”, to reflect the ethnicities of the depicted football players, as well as “Middle-Eastern”, and “Mixed-Ethnicity”. In addition, observers were permitted to enter an alternative classification in text. For age, each option covered a period of ten years, with 10–19 years and 60 + years being the youngest and oldest possible responses, respectively. In addition, each question included “*Cannot tell*” as a possible response option. Finally, as a familiarity check, participants were also asked to name targets, or to provide unique semantic information, for any targets that were recognized.

## Results

The familiarity check indicated that none of the participants recognized any of the 14 targets. The mean percentage of correct responses was then calculated for the drone and high-quality images for the sex, race, and age decisions. An independent-samples t-test showed that the accuracy of sex decisions was better for high-quality face photographs at 98.6% (SD = 3.5) than the drone images at 62.6% (SD = 13.6), *t*(58) = 14.03, *p* < 0.001. A similar advantage for high-quality photographs was observed for race decisions, at 74.3% (SD = 14.8) versus 42.4% (SD = 10.3), *t*(58) = 9.68, *p* < 0.001, and age decisions at 46.4% (SD = 12.4) versus 26.9% (SD = 13.5), *t*(58) = 5.84, *p* < 0.001.

## Discussion

This experiment provides broader evidence that drone-captured footage forms an unreliable basis for person perception. Sex information, for instance, was extracted poorly from drone stills, for which only 63% of targets were classified correctly. We did not plan to examine sex categorisation when we initiated this series of experiments, but were led to examine this question by the poor identification accuracy in Experiments 1 to 3. Consequently, all of the targets in the drone-captured footage were men, rather than a mixture of males and females. This one-sided sample could have affected observers’ responses from drone-captured footage, by leading to some female-sex decisions on the basis that observers might have expected a proportion of such stimuli in a sex categorization task. However, this was clearly not the case for the high-quality face photographs, for which performance was at ceiling. This contrast demonstrates that the low accuracy of sex categorization is reflective of the drone-captured footage, rather than the composition of targets’ sexes in this experiment. Accuracy for race and age decisions from drone-captured footage was even lower, at 42% and 27%, respectively. Thus, these decisions were more likely to be incorrect than correct under the current conditions. By contrast, performance with the face photographs here, as well as previous research, demonstrates that sex, race and age information are consistently extracted with much better accuracy from high-quality images^50–54^.

## General Discussion

This study explored the extent to which people can be identified from aerial footage recorded by a remote-controlled drone. The identities of unfamiliar (Experiment 1) and familiar target people (Experiment 2 and 3), and their sex, age and race (Experiment 4) were difficult to extract from drone-captured footage. This suggests that such footage provides a challenging substrate for person classification. In an extension of this work, we also compared identification of unfamiliar (Experiment 1) and familiar targets (Experiment 2) for footage from two different camera types, comprising the drone’s integrated HD camera and a retro-fitted GoPro. Whilst this revealed a small advantage for the drone’s integrated camera, identification accuracy was generally low for both camera types.

Many factors could account for these results. The movement speed and trajectory of the drone, its flight stability, as well as distance-to-target and its high vantage point are likely to degrade the available information for person identification. On the other hand, we employed high-definition recording equipment with good image stabilisation, flight height was limited to only 15 meters, targets were recorded from pitch-side, against a uniform background (the green pitch), and the number of (familiar) target identities was limited.

In this context, the current data have important implications. Drones are already employed routinely in military and police operations, for example, in searches for missing persons^1^, crowd control^2^, and military reconnaissance and lethal strikes^3,4^. The drone of the current study provides a limited proxy for military aircraft drones. However, some of the smaller drones employed by military and police are comparable to the equipment of this study^6^. For example, some police-employed drones operate at altitudes between ground level and 400 ft and at speeds from 0 to 38 kts^3,6,7^. Moreover, some of these drones carry recording equipment with a resolution that is substantially less than the cameras employed here (e.g., only 640 × 512 pixels)^55^. By comparison, the drone in the current study recorded targets from a maximum altitude of 49 ft in the experiment and was equipped with two cameras with considerably higher resolution (e.g., 1280 × 720 pixels for the drone’s integrated HD camera). In addition, the targets’ distance and orientation to the drone varied naturally during the recordings, thereby providing multiple perspectives to facilitate identification. These advantages did not appear to offset the difficulty of the task, however. Consequently, the finding that it is extremely difficult to identify people, or even just their sex, race and age from drone-recorded footage, such as that provided in the current study, raises concerns about their application for person perception in police and military operations.

In drawing these conclusions, we note also that this is only the first study to explore person identification with drone-captured footage. Our trial count was restricted by the number of targets that could be recorded, whilst sample size was limited by participants who were familiar with these targets. Moreover, it is presently unknown how these findings generalize across different drone types and viewing conditions. It is possible, for example, that the poor accuracy in person identification that was observed here can be offset when flight height is lowered or drone-to-target distance is reduced, though operational requirements may not allow this to safeguard those on the ground^56^ or to avoid detection of a drone during covert deployment^2^. Similarly, it is possible that person identification from drone-captured footage might be improved by magnification equipment, such as optical zoom, though this may also increase the difficulty of target tracking. In addition, we note that the identification of unfamiliar people can be difficult even with high-quality face portraits^8,26,28,42^. Thus, the extent to which person identification is possible from drone-captured footage under viewing conditions that are optimised further is an open question.

Finally, whilst the aim of the current study was to examine person identification from drone-captured footage by human observers, similar studies in computer vision are now beginning to emerge^57^. This raises the question of how the accuracy of human observers and machine algorithms in person identification might compare. Face-matching studies with more conventional footage suggest that machine algorithms outperform human observers under conditions of moderate difficulty^58,59^, and perform at least to a similar level with challenging face pairs, such as images in which illumination and a person’s day-to-day appearance are variable^58,59^. However, the face images that were employed in these studies are of substantially higher quality than the drone-footage under investigation here, making it difficult to draw direct comparisons at this point in time.

In conclusion, the current study suggests that a person’s identity, sex, race and age are difficult to extract from drone-captured footage. However, more extensive studies are clearly needed to investigate unfamiliar and familiar person identification and categorization from drone-captured footage, with more stimuli and greater sample sizes, utilising more drone types, a greater range of image-capture and magnification devices, and with footage recorded under a much wider range of viewing conditions. Comparisons of human observers and machine algorithms in person identification from drone-captured footage are also required to advance the field.
